# Development of species-specific SCAR markers for identification and authentication of three rare Peninsular Malaysian endemic *Coelogyne *(Orchidaceae) orchids

**DOI:** 10.12688/f1000research.26170.2

**Published:** 2021-01-05

**Authors:** Yoh Kok Hon, Christina Seok-Yien Yong, Janna Ong Abdullah, Rusea Go

**Affiliations:** 1 Department of Biology, Faculty of Science, Universiti Putra Malaysia, Serdang, Selangor, 43400, Malaysia; 2 Department of Cell and Molecular Biology, Faculty of Biotechnology and Biomolecular Sciences, Universiti Putra Malaysia, Serdang, Selangor, 43400, Malaysia

**Keywords:** Coelogyne kaliana, Coelogyne stenochila, Coelogyne tiomanensis, endemic species, RAPD, SCAR, species identification, taxon delimitation

## Abstract

**Background:** *Coelogyne kaliana*, *Coelogyne stenochila* and *Coelogyne tiomanensis *are three valuable rare orchid species endemic to Peninsular Malaysia, currently rampantly traded illegally via the internet and through local nurseries, which label them as hybrids to avoid enforcement detection. Drastic measures to ensure the continued existence of their populations in the wild should be introduced as they are rapidly diminishing into extinction, including the development of rapid and accurate species-specific identification tools. These three orchid species are highly similar morphologically and currently it is impossible to distinguish among them without their reproductive structures.

**Methods: ** RAPD-based species-specific SCAR markers were developed to distinguish and authenticate the identity of these three endemic Peninsular Malaysian *Coelogyne* species.

**Results: **Three SCAR markers were successfully developed in this study. SCAR marker primer pair **, CKL_f / CKL_r** was specific to *C. kaliana *as it produced a unique single band of 271 bp but not in C. *stenochila *and *C. tiomanensis*.  SCAR marker primer pair **CST_f / CST_r** amplified a single band of 854 bp in *C. stenochila* and two bands of different sizes (372 bp and 858 bp) in *C. tiomanensis, *but no amplification in *C. kaliana*. The third SCAR marker primer pair, **CTI_f / CTI_r **produced a single band (about 500 bp) for both *C. stenochila* and *C. tiomanensis,* but showed no amplification in *C. kaliana*.

**Conclusions: **Although not all these SCAR markers were species amplification specific, they could be used to discriminate among the three *Coelogyne *species effectively.  Accurate species identification is one of the most important steps to allow a proper management plan to be established in the effort to conserve these three endangered orchid species of Peninsular Malaysia. Besides, it could effectively put a stop to the illegal trading of these rare endangered orchid species worldwide.

## Introduction

**Figure 1.**
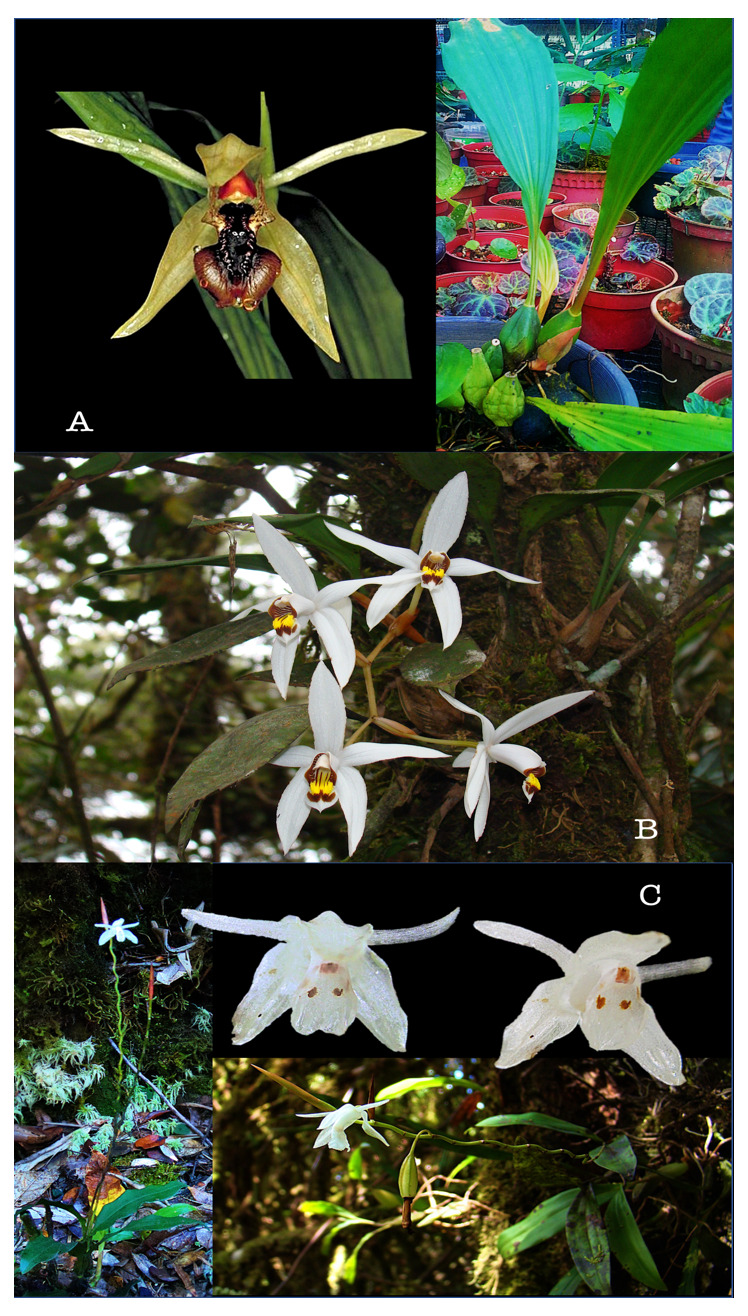
The three endemic *Coelogyne* species in Peninsular Malaysia. ( **A**) *Coelogyne tiomanensis;* ( **B**) *Coelogyne kaliana* and ( **C**) *Coelogyne stenochila* (all with flower and vegetative structure).

The genus *Coelogyne* L. belongs to the Orchidaceae family, which is comprised of about 200 sympodial species distributed throughout India, southwest China, southeast Asia and the Fiji Islands. Their main centres of diversity are Borneo, Papua New Guinea, Sumatra and the Himalayan range ( Butzin, 1992; Gravendeel *et al.,* 2001). There are 26 species of *Coelogyne* in Peninsular Malaysia based on the latest Checklist of Orchids of Peninsular Malaysia ( Ong *et al.,* 2017) and Seidenfaden & Wood, 1992. Amongst them, *Coelogyne kaliana*, *Coelogyne stenochila* and *Coelogyne tiomanensis* are endemic to Peninsular Malaysia ( Figure 1). They are mostly found in the highland regions at elevations of 1200 m above sea level. These three Peninsular Malaysian endemic species are very rare and are present in small populations. Endemic orchid species are national treasures, which have high commercial values among orchid collectors and enthusiasts. This leads to their illegal and indiscriminate collections from the wild. Moreover, the survival of many endemic species may be jeopardized due to rapid climate change, which causes their populations to decline gradually. This eventually would reduce the likelihood of them to being found in their natural habitats ( Ong, 2013). Scientific and efficient discriminations of morphologically similar species have been indispensable in achieving the ultimate goal of preservation and conservation of endemic orchid species. Apart from their distinctive floral appearances, *C. kaliana*, *C. stenochila* and *C. tiomanensis* are morphologically similar with indistinguishable common vegetative structures. Thus, it is crucial that molecular markers be developed, which would enable us to easily distinguish among them taxonomically.

Random Amplified Polymorphic DNA (RAPD) is a polymerase chain reaction (PCR)-based method ( Williams *et al.,* 1990) that uses short and arbitrary oligonucleotides with GC contents of at least 50% to produce amplification products at random. The short oligonucleotide primer of approximately 10 bp in length is called a decamer and serves as both the forward and reverse primers. The amplification process in RAPD analysis is performed on total genomic DNA. Thus, it can be used to study genetic polymorphisms within the whole genome instead of just polymorphisms within a single genetic region. It also allows the amplification of random fragments of the genome without prior sequence knowledge. The main constraint of RAPD is its low fidelity. However, the conversion of RAPD markers into new, longer and specific Sequence Characterized Amplified Region (SCAR) markers can significantly improve reliability and reproducibility. Some other PCR-based methods, such as single locus microsatellites, Inter-Simple Sequence Repeats (ISSR) and Amplified Fragment Length Polymorphisms (AFLP), show better reproducibility of amplifications than the RAPD method. But, SCAR marker developments from these methods are often costlier, time consuming and laborious ( Kumar & Gurusubramanian, 2011) when compared to its development from the RAPD method.

The SCAR marker ( Paran & Michelmore, 1993) is a robust and reliable method used to detect and differentiate different samples by using specific primers derived from RAPD, ISSR, AFLP and other DNA markers. SCAR markers can discriminate among closely related samples or species by amplifying products of different sizes or no amplification in non-targeted samples and positive amplifications in targeted samples. Over the past few years, this method had been widely adopted in the identification of morphologically similar but genetically different organisms. A large number of robust and reliable SCAR markers had been successfully developed through the RAPD-based method for *Pisum sativum* ( Srivastava *et al.,* 2012), *Oryza sativa* ( Semsang *et al.,* 2013), *Sorghum halepense* ( Zhang *et al.,* 2013) and even for orchids, such as *Phalaenopsis* ( Goh *et al.,* 2005; Niknejad *et al.,* 2009) and *Paphiopedilum* ( Sun *et al.,* 2011). However, currently there is still no genetic marker for the species identification of Peninsular Malaysia’s endemic *Coelogyne* species.

Hence, in this present investigation, RAPD-based SCAR markers were developed to distinguish among the three endemic *Coelogyne* species in Peninsular Malaysia. RAPD primers were used to screen the DNA samples of these three species to amplify reproducible species-specific bands. A single or a combination of two SCAR markers, which we developed, allowed us to successfully distinguish among the three morphologically similar and closely related Peninsular Malaysian endemic *Coelogyne* species. To our best knowledge, this is the first study on developing species-specific SCAR markers to identify the three endemic *Coelogyne* orchids of Peninsular Malaysia.

## Methods

### Plant materials and DNA extraction

Due to the rarity of these species, only two samples of *C. kaliana* (YKH 020, Genting Highlands and YKH 004, Cameron Highlands, Malaysia), one sample of *C. stenochila* (YKH 031, Gunung Tahan, Malaysia) and one sample of *C. tiomanensis* (FRI 75329, Tioman Island, Malaysia) were collected from their native locations in Peninsular Malaysia. A small piece (3 cm × 3 cm) of fresh leaf from each sample was used for the DNA extraction. Genomic DNA was extracted based on the conventional CTAB method ( Doyle & Doyle, 1987). The DNA pellets were suspended in 50 μl sterile DNase free water and kept at −20 °C until ready for use. The purity and concentration of the DNA samples were determined using a spectrophotometer (NanoDrop, USA).

### RAPD-PCR amplification

A total of 16 decamer universal RAPD primers (synthesized by First Base Laboratory, Serdang, Malaysia), as shown in Table 1, were used to amplify the DNA samples of the three Malaysian endemic *Coelogyne* species. These sequences were obtained from published articles ( Table 1) where they have been used successfully in other orchid species. The RAPD amplification reactions were performed in a 10 μl volume containing 50 ng of plant DNA, 1.0 μM of RAPD primer (First Base Laboratory), 1 × PCR buffer (Promega, USA), 2.5 mM of MgCl _2_ (Promega, USA), 0.1mM dNTPs mix (Promega, USA), 0.5 units of Taq DNA polymerase (Promega, USA), using a thermal cycler machine (Eppendorf Master Cycler Gradient, Hamburg, Germany), The run conditions were: 1 cycle of initial denaturation at 95°C for 10 min; 45 cycles of denaturation at 95°C for 1 min, annealing at 35°C for 2 min and extension at 72°C for 2 min; and 1 cycle of final extension at 72°C for 10 min. The amplicons were electrophoresed on 1.2% TBE (Tris-borate-EDTA) agarose gel for 90 minutes at 70 V and stained with 0.5μg/mL ethidium bromide. The gel was visualised under a UV transilluminator (UVIdoc, USA) and the gel image was taken using a camera (UVItec, UK). Species-specific bands were identified from the bands produced during the RAPD amplifications.

**Table 1.** RAPD primers used for PCR amplifications.

Primer code	Sequence 5'−3'	Reference
OPA 03	AGTCAGCCAC	( Grünanger *et al.,* 1998; Obara-Okeyo & Kako, 1998; Parab & Krishnan, 2008)
OPA 04	AATCGGGCTG	( Parab & Krishnan, 2008)
OPA 09	GGGTAACGCC	( Grünanger *et al.,* 1998; Parab & Krishnan, 2008)
OPA 12	TCGGCGATAG	( Grünanger *et al.,* 1998; Parab & Krishnan, 2008)
OPA 14	TCTGTGCTGG	( Grünanger *et al.,* 1998; Parab & Krishnan, 2008)
OPF 02	GAGGATCCCT	( Benner *et al.,* 1995)
OPF 03	CCTGATCACC	( Benner *et al.,* 1995)
OPF 04	GGTGATCAGG	( Benner *et al.,* 1995)
OPF 05	CCGAATTCCC	( Benner *et al.,* 1995)
OPF 06	GGGAATTCGG	( Benner *et al.,* 1995)
OPU 03	CTATGCCGAC	( Goh *et al.,* 2005; Lim *et al.,* 1999; Niknejad *et al.,* 2009)
OPU 08	GGCGAAGGTT	( Goh *et al.,* 2005; Niknejad *et al.,* 2009)
OPU 10	ACCTCGGCAC	( Goh *et al.,* 2005; Niknejad *et al.,* 2009)
OPU 12	TCACCAGCCA	( Goh *et al.,* 2005; Niknejad *et al.,* 2009)
OPU 13	GGCTGGTTCC	Goh *et al.*, 2005; Niknejad *et al.*, 2009
OPU 16	CTGCGCTGGA	Goh *et al.*, 2005; Niknejad *et al.*, 2009

### Cloning and sequencing of specific RAPD fragments

Primers that consistently generated species specific PCR profiles in all the DNA samples were selected for primer design. The amplified PCR products were cloned and sequenced using the service provided by First Base Laboratory. All species-specific RAPD-generated PCR amplicons were cloned into pJET1.2/blunt cloning vector by chemical transformation into the *E. coli* competent cells. The white colonies, which contained the recombinant DNA (plasmid), were picked from LB/ampicillin/X-gal plates and the recombinant DNA was isolated from the bacterial culture. The targeted inserts were sequenced using SP6 and T7 universal sequencing primers.

### Design and validation of SCAR primers

The DNA sequences obtained were first subjected to nucleotide similarity searches using the BLASTN function of the NCBI database to check for significant similarities of the sequences with any sequences in GenBank Species-specific SCAR marker primer pairs were then designed based on the DNA sequences obtained. Species-specific SCAR marker primer pairs of 20–24 bases in length were designed with high stringency using the program Primer3 ( Rozen & Skaletsky, 2000). Then, the designed SCAR marker primer pairs were synthesised by First Base Laboratory. Primer specificities were validated by amplifying the SCAR markers using the DNA samples of the three endemic *Coelogyne* species and by the same reaction mixture as was described in the section *RAPD-PCR Amplification*. The PCR profiles used for the amplifications of the designed SCAR primer pairs were: initial denaturation at 95°C for 10 min, 35 cycles of denaturation at 95°C for 1 min, annealing at 51 to 53°C (depending on the SCAR primer pair) for 2 min, extension at 72°C for 2 min, and a final extension cycle at 72°C for 10 min. The amplified PCR products were sequenced by First Base Laboratory, Serdang, Malaysia to confirm sequence specificities.

## Results

### RAPD profile analysis

**Figure 2.**
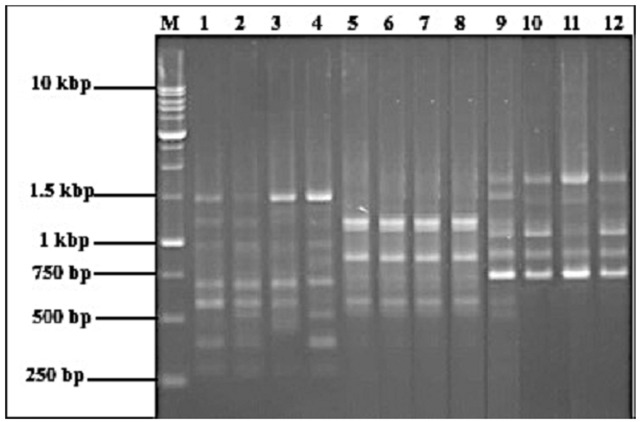
RAPD fingerprint results from amplification of DNA samples using primers OPU 08 on 1.2% agarose gel. Lanes 1 and 2 are biological samples of *C. kaliana* (YKH004 and YKH020) while lanes 3 and 4 are technical replicates of YKH004 and YKH020 respectively; lane 5 is a biological sample of *C. stenochila* (YKH031) while lanes 6 to 8 are technical replicates of YKH031; lane 9 is a biological sample of *C. tiomanensis* (FRI75329) while lanes 10 to 12 are technical replicates of FRI75329. Lane M represents the 1.0 kb DNA ladder (Promega, USA). Fragments indicated by arrows were the specific bands, which were selected and subsequently sequenced for SCAR marker primer pair design.

In the preliminary screenings of 16 random decanucleotide RAPD primers, all primers were able to amplify genomic DNA samples of *Coelogyne kaliana*, *Coelogyne stenochila* and *Coelogyne tiomanensis*, to produce a variable number of bands of different sizes. However, out of the 16 universal RAPD primers screened, only two RAPD primers, namely OPU 08 and OPU 12 showed a high level of consistency, producing distinct and reproducible fingerprint patterns in all the DNA samples tested. Amplification by primer OPU 08 consistently produced a clear band between 1 kb and 1.5 kb ( Figure 2), which was unique to *C. stenochila* but absent in *C. kaliana* and *C. tiomanensis*. Primer OPU 08 also consistently amplified an intense band of roughly 750 bp that was specific to *C. tiomanensis* only and not found in *C. stenochila* and *C. kaliana*. Primer OPU 12 consistently amplified a distinct fragment of about 500 bp ( Figure 2), which was present in all DNA samples of *C. kaliana,* but which was not observed in the DNA samples of *C. stenochila* and *C. tiomanensis*. 

**Figure 3.**
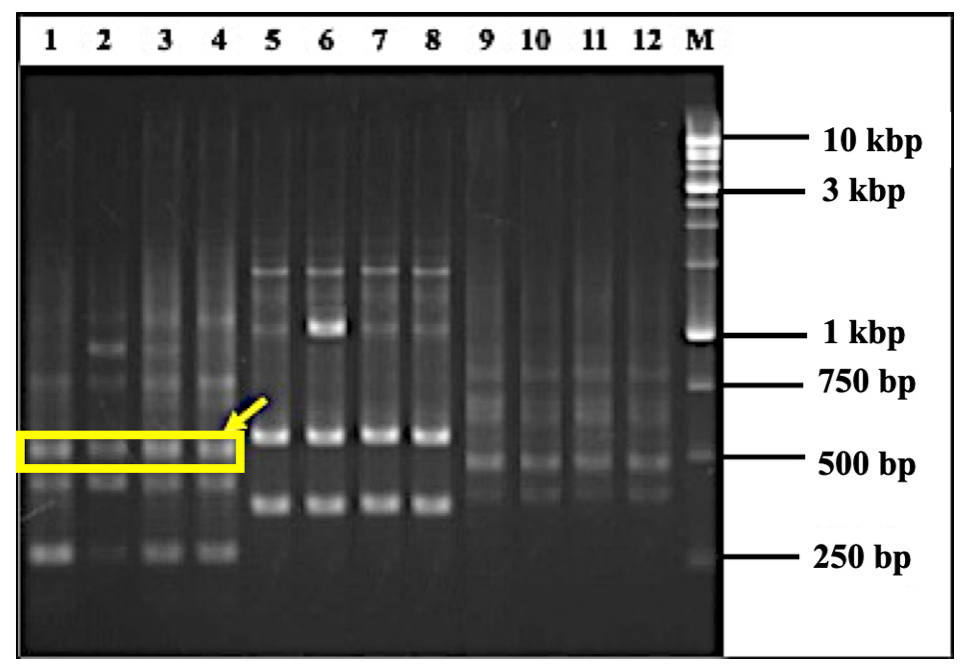
RAPD fingerprints amplified from DNA samples using primers OPU 12 on 1.2% agarose gel. Lanes 1 and 2 are biological samples while lanes 3 and 4 are technical replicates of *C. kaliana*; lane 5 is a biological sample while lanes 6 to 8 are technical replicates of *C. stenochila*; lane 9 is a biological sample while lanes 10 to 12 are technical replicates of *C. tiomanensis*. Lane M represents the 1.0 kb DNA ladder (Promega, USA). Fragments indicated by arrows were the specific bands selected and sequenced for SCAR marker primer pair design.

Based on the RAPD fingerprinting results of OPU 08 and OPU 12, very few species-specific bands were observed. However, in selecting species-specific fragments for cloning and sequencing, other than focusing on bright and clear monomorphic band, fragment size was also taken into consideration. The ideal fragment size usually should range from 500 bp to 1.5 kbp, as too large a fragment faced difficulty in the cloning process; while too small a fragment provided less sequence for designing the SCAR marker primer pair. Therefore, a fragment of between 1 kbp to 1.5 kbp amplified by the OPU 08 primer was selected for *C. stenochila,* while another of approximately 750 bp was selected for *C. tiomanensis*. A fragment of about 500 bp amplified by primer OPU 12 was chosen for *C. kaliana* ( Figure 3). 

Sequencing of the selected fragments yielded a sequence of 517 bp for *C. kaliana*, 1207 bp for *C. stenochila* and 742 bp for *C. tiomanensis* respectively. Homology searches using BLASTN showed that the fragments did not have similarity with any known nucleotide sequences in the NCBI database. The SCAR primer pairs of 20 to 24 bases each were designed with high stringency to ensure the specificity for each endemic *Coelogyne* species using these primer pair sequences. Details of the three designed primer pairs are shown in Table 2.

**Table 2.** Species-specific SCAR primer pairs derived from RAPD amplified nucleotide sequences for *C. kaliana*, *C. stenochila* and *C. tiomanensis*.

Species	Locality	Section	Voucher	Gene bank accession number	Collector name	Date of collection	SCAR primer name	Sequence (5’-3’)	Annealing tempeature (°C)	Expected size(bp)
***C.kaliana***	Genting Highlands, Malaysia	Tomentosae	YKH 004	MK398165	Yoh Kok Hon & Rusea Go (UPM)	10 Jan. 2020	CKL_f CKL_r	AGTGGAAAATCCGTTCAGTCTC TCAAAACCTCAGTCCCTCTTC	51.7	297
***C.kaliana***	Cameron Highlands, Malaysia	Tomentosae	YKH 020	MK398164	Yoh Kok Hon & Rusea Go (UPM)	14 Feb. 2020	CKL_f CKL_r	AGTGGAAAATCCGTTCAGTCTC TCAAAACCTCAGTCCCTCTTC	51.7	297
***C. stenochila***	Gunung Tahan, Malaysia	Longifoliae	YKH 031	MK398181	Yoh Kok Hon & Rusea Go (UPM)	3 Sept. 2013	CST_f CST_r	ACTATGTTGGTTAGGCGGTG CTTTTCTGAGAGAGGGGTGT	51.6	874
***C. tiomanensis***	Gunung Kajang, Malaysia	Speciosae	FRI 75329	MK398182	Ong Poh Tech (FRIM)	8 Aug. 2013	CTI_f CTI_r	AGGAGGAAATCACGAGCATAACA TGCGTTGAAAATAGCAGGAGCG	53.2	591

**Figure 4.**
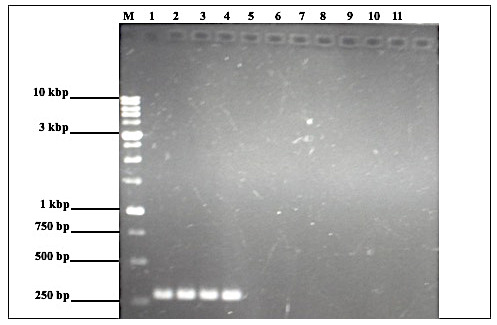
PCR amplification of genomic DNA from *C. kaliana* (lanes 1 to 4), *C. stenochila* (lanes 5 to 7) and *C. tiomanensis* (lanes 8 to 11) using the designed SCAR primer pair (CKL_f / CKL_r). Lane M represents the 1.0 kb DNA ladder (Promega, USA).

The efficacy and specificity of the designed SCAR primers were screened for using DNA samples of the three endemic *Coelogyne* species. SCAR primer pair CKL_f and CKL_r amplified a single, clear, distinct and easily identifiable band of 271 bp only in DNA samples of *C. kaliana*, while no amplification was observed for *C. stenochila* and *C. tiomanensis* ( Figure 4). Hence, the SCAR primer pair CKL_f and CKL_r is a species-specific marker for *C. kaliana*.

**Figure 5.**
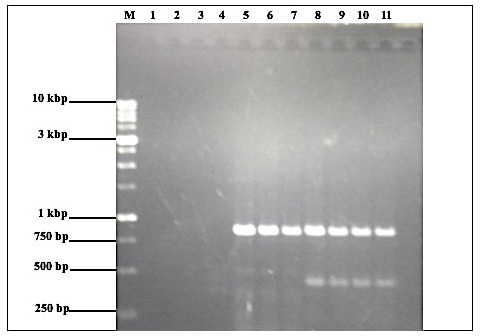
PCR amplification of genomic DNA from *C. kaliana* (lanes 1 to 4), *C. stenochila* (lanes 5 to 7) and *C. tiomanensis* (lanes 8 to 11) using the designed SCAR primer pair (CST_f / CST_r). Lane M represents the 1.0 kb DNA ladder (Promega, USA).

The SCAR primer pair CST_f and CST_r, designed based on the specific fragment selected from the RAPD profile of *C*. *stenochila*, was not species amplification specific as anticipated. However, this SCAR marker still allowed the differentiation of the three endemic *Coelogyne* species by amplifying a single and bright band of 854 bp in *C. stenochila* but two bands of different sizes (858bp and 372 bp) in *C. tiomanensis*, and no DNA amplification in *C. kaliana* ( Figure 5). 

**Figure 6.**
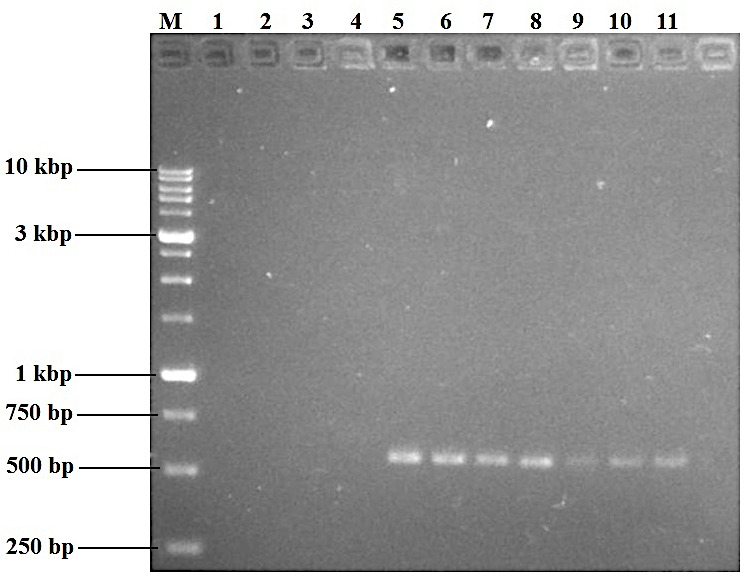
PCR amplification of genomic DNA from *C. kaliana* (lanes 1 to 4), *C. stenochila* (lanes 5 to 7) and *C. tiomanensis* (lanes 8 to 11) using the designed SCAR primer pair (CTI_f / CTI_r). Lane M represents the 1.0 kb DNA ladder (Promega, USA).

The SCAR primer pair CTI_f and CTI_r, designed from the species-specific RAPD fragment for *C. tiomanensis*, was also not species amplification specific to *C. tiomanensis*. Nonetheless, it could still be used to differentiate *C. kaliana* from the other two endemic species, *C. stenochila* and *C. tiomanensis.* This SCAR marker amplified a single band of about 500 bp in both *C. stenochila* and *C. tiomanensis* but no DNA amplification in *C. kaliana* ( Figure 6).

## Discussion

Based on the results of the present study, selected bands amplified by universal RAPD primers were successfully converted into specific SCAR markers. These SCAR markers were able to be used for the rapid identification and authentication of three Peninsular Malaysian endemic *Coelogyne* species, *Coelogyne kaliana*, *Coelogyne stenochila* and *Coelogyne tiomanensis*. Endemic species refers to species that are found exclusively or confined to a restricted geographical area. Both *Coelogyne kaliana* and *Coelogyne tiomanensis* were named after the regions from which they were first found. *Coelogyne kaliana* was first discovered in Gunung Ulu Kali of Genting Highlands in Peninsular Malaysia and was described by Cribb in 1982. *Coelogyne tiomanensis,* first described by Henderson in 1930, is found exclusively in Gunung Kajang of Tioman Island. *Coelogyne stenochila*, first described by Hook in 1890, can only be found in Pahang and Selangor states ( Seidenfaden & Wood, 1992). The endemism and rarity of these *Coelogyne* species make their conservation of utmost importance. The accurate identification of these three morphologically similar species is one of the first steps towards achieving this goal by effectively preventing their illegal trading. 

To the best of our knowledge, this is the first reported RAPD-SCAR study on the three endemic Peninsular Malaysian *Coelogyne* species. Although not all the SCAR markers developed in this study were species amplification specific, they could still elucidate the identity of the three endemic *Coelogyne* species efficiently and correctly. This was impossible through the traditional vegetative morphological characteristics method usually used by botanists. The main constraint in this study was that the number of samples available for use was very few due to the rarity and protected status of these endemic species. Thus, a comprehensive screening on more samples of the three species was not feasible in this case. Furthermore, the RAPD-SCAR markers have yet to be tested on other Malaysian *Coelogyne* to confirm the specificity of the markers. Hence, the RADP-SCAR markers should be tested on the more commonly available *Coelogyne* species such as *C. asperata and C. speciosa* in future experiments. Nonetheless, the SCAR markers developed in this study are new to science and are able to differentiate the three species accurately. This study further reinforced the robustness and suitability of SCAR makers as one of the leading methods for species identification of members of cryptic species complexes. This approach could also be used for other flagship species of economic and aesthetic importance. We hope that with the assistance of these markers, an exhaustive conservation plan could be developed in the immediate future to ensure the continued survival of these three rare endemic orchid species. Currently, the biggest threat to their survival is the high demand for them by orchid enthusiasts worldwide. This has resulted in them being obtained and traded illegally often by the sellers claiming that they are selling hybrid plants. It is expected that with the availability of the SCAR markers, which we have developed, this legal loophole can be successfully plugged.

## Data availability

### Underlying data

Sequences are available from GenBank:

- Coelogyne kaliana voucher YKH 004 maturase K (matK) gene, partial cds; chloroplast, Accession number MK398222.1: https://www.ncbi.nlm.nih.gov/nuccore/MK398222.1
- Coelogyne kaliana voucher YKH 020 maturase K (matK) gene, partial cds; chloroplast, Accession number MK398221.1: https://www.ncbi.nlm.nih.gov/nuccore/MK398221.1
- Coelogyne kaliana voucher YKH 004 ribulose-1,5-bisphosphate carboxylase/oxygenase large subunit (rbcL) gene, partial cds; chloroplast, Accession number MK398165.1: https://www.ncbi.nlm.nih.gov/nuccore/MK398165.1
- Coelogyne kaliana voucher YKH 020 ribulose-1,5-bisphosphate carboxylase/oxygenase large subunit (rbcL) gene, partial cds; chloroplast, Accession number MK398164.1:
- Coelogyne kaliana voucher YKH 004 tRNA-Leu (trnL) gene and trnL-trnF intergenic spacer, partial sequence; chloroplast, Accession number MK356248.1: https://www.ncbi.nlm.nih.gov/nuccore/MK356248.1
- Coelogyne kaliana voucher YKH 004 small subunit ribosomal RNA gene, partial sequence; internal transcribed spacer 1, 5.8S ribosomal RNA gene, and internal transcribed spacer 2, complete sequence; and large subunit ribosomal RNA gene, partial sequence, Accession number MK356194.1: https://www.ncbi.nlm.nih.gov/nuccore/MK356194.1
- Coelogyne kaliana voucher YKH 020 small subunit ribosomal RNA gene, partial sequence; internal transcribed spacer 1, 5.8S ribosomal RNA gene, and internal transcribed spacer 2, complete sequence; and large subunit ribosomal RNA gene, partial sequence, Accession number MK356193.1: https://www.ncbi.nlm.nih.gov/nuccore/MK356193.1
- Coelogyne kaliana voucher YKH 020 tRNA-Leu (trnL) gene and trnL-trnF intergenic spacer, partial sequence; chloroplast, Accession number MK356247.1: https://www.ncbi.nlm.nih.gov/nuccore/MK356247.1
- Coelogyne tiomanensis voucher FRI 75329 maturase K (matK) gene, partial cds; chloroplast, Accession number MK398239.1: https://www.ncbi.nlm.nih.gov/nuccore/MK398239.1
- Coelogyne tiomanensis voucher FRI 75329 small subunit ribosomal RNA gene, partial sequence; internal transcribed spacer 1, 5.8S ribosomal RNA gene, and internal transcribed spacer 2, complete sequence; and large subunit ribosomal RNA gene, partial sequence, Accession number MK356154.1: https://www.ncbi.nlm.nih.gov/nuccore/MK356154.1
- Coelogyne tiomanensis tRNA-Leu (trnL) gene and trnL-trnF intergenic spacer, partial sequence; chloroplast, Accession number MK356265.1: https://www.ncbi.nlm.nih.gov/nuccore/MK356265.1
- Coelogyne tiomanensis voucher FRI 75329 ribulose-1,5-bisphosphate carboxylase/oxygenase large subunit (rbcL) gene, partial cds; chloroplast, Accession number MK398182.1: https://www.ncbi.nlm.nih.gov/nuccore/MK398182.1
- Coelogyne stenochila voucher YKH 031 ribulose-1,5-bisphosphate carboxylase/oxygenase large subunit (rbcL) gene, partial cds; chloroplast, Accession number MK398181.1: https://www.ncbi.nlm.nih.gov/nuccore/MK398181.1
- Coelogyne stenochila voucher YKH 031 small subunit ribosomal RNA gene, partial sequence; internal transcribed spacer 1, 5.8S ribosomal RNA gene, and internal transcribed spacer 2, complete sequence; and large subunit ribosomal RNA gene, partial sequence, Accession number MK356153.1:
- Coelogyne stenochila voucher YKH 031 maturase K (matK) gene, partial cds; chloroplast, Accession number MK398238.1:
- Coelogyne stenochila tRNA-Leu (trnL) gene and trnL-trnF intergenic spacer, partial sequence; chloroplast, Accession number MK356264.1: https://www.ncbi.nlm.nih.gov/nuccore/MK356264.1

Open Science Framework: Development of species-specific SCAR markers for identification and authentication of three rare Peninsular Malaysian endemic Coelogyne (Orchidaceae) orchids, http://doi.org/10.17605/OSF.IO/35XBG ( Go, 2020) (project registered on September 1st, 2020; https://osf.io/dxtb2). 

This project contains the following underlying data:

- Sequences resulted from cloning.
- Uncropped/unedited images of RAPD fingerprint blots for the 16 decamer universal RAPD primers.
- Uncropped/unedited images of SCAR primer pair blots.

Data are available under the terms of the Creative Commons Attribution 4.0 International license (CC-BY 4.0). 
