# Supplementary material for: Development of species-specific SCAR markers for identification and authentication of three rare Peninsular Malaysian endemic Coelogyne (Orchidaceae) orchids
Source: F1000Res. 2021 Jan 5;9:1161. Originally published 2020 Sep 21. [Version 2] doi: 10.12688/f1000research.26170.2 (PMC7707113; doi:10.12688/f1000research.26170.2)
Supplement: Peer review history

# Peer review: Reviewer response for version 2

**DOI:** 10.5256/f1000research.31486.r76641

**Published:** 2021-01-06

**Authors:** Jean Wan Hong Yong

**Affiliations:** 1 Department of Biosystems and Technology, Swedish University of Agricultural Sciences, Alnarp, Sweden

The revised paper has addressed all the suggestions and clarifications to improve the manuscript. Thus, it is suitable for indexing in the Journal

Is the work clearly and accurately presented and does it cite the current literature?

Yes

If applicable, is the statistical analysis and its interpretation appropriate?

Not applicable

Are all the source data underlying the results available to ensure full reproducibility?

Yes

Is the study design appropriate and is the work technically sound?

Yes

Are the conclusions drawn adequately supported by the results?

Yes

Are sufficient details of methods and analysis provided to allow replication by others?

Yes

Reviewer Expertise:

Orchid biology, Horticulture

I confirm that I have read this submission and believe that I have an appropriate level of expertise to confirm that it is of an acceptable scientific standard.

# Peer review: Reviewer response for version 1

**DOI:** 10.5256/f1000research.28880.r71757

**Published:** 2020-11-30

**Authors:** Jean Wan Hong Yong

**Affiliations:** 1 Department of Biosystems and Technology, Swedish University of Agricultural Sciences, Alnarp, Sweden

This is an interesting, and timely paper pertaining to the molecular identification of three endemic *Coelogyne* (Orchidaceae) orchids in Malaysia. When these orchids are not flowering (vegetative stage), it is nearly impossible to identify them taxonomically.

 Sequence Characterized Amplified Region (SCAR) markers, an improvement from RAPD, was used to discriminate successfully the three closely related *Coelogyne* species in Peninsular Malaysia.

 The literature review carried out by the authors was adequate, appropriate, and organized in a logical fashion. Only minor adjustment is needed (see below).

 The paper was well written and the experiments were conducted carefully. The sample size, research approaches (standard molecular techniques, following manufacturer’s protocols) and profile analyses, were appropriate.

**Scientific suggestion**

 My only technical critique is the lack of “outlier” in the analyses. The type species is *Coelogyne cristata* (may not be available to the authors). Perhaps, the authors could consider using a commonly available Malaysian *Coelogyne* (e.g. *C. asperata*) as an ‘“outlier” in future experiments as a form of control. Hence, the authors could discuss/explain the lack of a “outlier” in their experiments.

**Figure improvement**

 A new figure 2 or an expanded Fig 1 with vegetative features (featuring leaf and pseudobulb) of the three *Coelogyne *species should be included, in addition to the (beautiful) flowers. See Plate 12 (four *Coelogyne* species featured, especially *C. viscosa*), page 732, The Orchids of Peninsular Malaysia and Singapore as a photographic guide ^1^.

**Text corrections**

 "Seidenfaden and Wood, (1992)" ^1^, the authoritative reference for orchids of Malaysia should feature prominently in the Introduction.

 There are 26 species of *Coelogyne* in Peninsular Malaysia based on the latest Checklist of Orchids of Peninsular Malaysia ^2^ (see also Seidenfaden and Wood, 1992) ^1^.

**Recommendation**

 This paper should be indexed with some minor revision.

Is the work clearly and accurately presented and does it cite the current literature?

Yes

If applicable, is the statistical analysis and its interpretation appropriate?

Not applicable

Are all the source data underlying the results available to ensure full reproducibility?

Yes

Is the study design appropriate and is the work technically sound?

Yes

Are the conclusions drawn adequately supported by the results?

Yes

Are sufficient details of methods and analysis provided to allow replication by others?

Yes

Reviewer Expertise:

Orchid biology

I confirm that I have read this submission and believe that I have an appropriate level of expertise to confirm that it is of an acceptable scientific standard.

## References

[1] : The Orchids of Peninsular Malaysia and Singapore.1992;

[2] : Checklist of Orchids of Peninsular Malaysia.2017;

# Peer review: Reviewer response for version 1

**DOI:** 10.5256/f1000research.28880.r71987

**Published:** 2020-10-13

**Authors:** Mohd Norfaizal Ghazali

**Affiliations:** 1 Resource Utilisation and Agrobiodiversity Conservation Programme (BE2), Agrobiodiversity and Environment Research Centre, Malaysian Agricultural Research and Development Institute (MARDI), Serdang, Malaysia

This manuscript discussed the information and identification of *Coelogyne kaliana, Coelogyne stenochila* and *Coelogyne tiomanensis* (Orchidaceae) which are considered as three unique rare orchid species. Although the SCAR markers used in this study were not considered as species amplification specific, the information is quite useful in discriminating three *Coelogyne* species effectively. This paper is recommended for your intended journal, and the authors are required to amend the following as suggested below:

**My recommendations:**

- Abstract section 

Insert Orchidaceae after *C. tiomanensis *(para 2)

 Keywords: *Coelogyne stenochila, C. tiomanensis, C. kaliniana* – italic 

- Methodology

Insert the plant materials collection and details in a **Table**

 The table should consist of these information:

 1. Date of collection

 2. Scientific name

 3. Localities

 4. Collector’s name

 5.Voucher number

- Discussion section

 Italic all the scientific name in the section:

**‘Data Availability’**

**Underlying data**

Is the work clearly and accurately presented and does it cite the current literature?

Yes

If applicable, is the statistical analysis and its interpretation appropriate?

Partly

Are all the source data underlying the results available to ensure full reproducibility?

Yes

Is the study design appropriate and is the work technically sound?

Yes

Are the conclusions drawn adequately supported by the results?

Yes

Are sufficient details of methods and analysis provided to allow replication by others?

Yes

Reviewer Expertise:

Plant Systematic, Plant Anatomy and Micromorphology, Palynology

I confirm that I have read this submission and believe that I have an appropriate level of expertise to confirm that it is of an acceptable scientific standard.
